# Structural, enzymatic and spatiotemporal regulation of PP2A-B55 phosphatase in the control of mitosis

**DOI:** 10.3389/fcell.2022.967909

**Published:** 2022-08-29

**Authors:** Benjamin Lacroix, Thierry Lorca, Anna Castro

**Affiliations:** ^1^ Centre de Recherche en Biologie Cellulaire de Montpellier (CRBM), CNRS UMR5237, Université de Montpellier, CNRS UMR5237Montpellier, France; ^2^ Équipe Labellisée “Ligue Nationale Contre le Cancer”, Paris, France

**Keywords:** PP2A phosphatase, cyclin B/Cdk1, greatwall, ARPP19, ENSA, mitosis

## Abstract

Cells require major physical changes to induce a proper repartition of the DNA. Nuclear envelope breakdown, DNA condensation and spindle formation are promoted at mitotic entry by massive protein phosphorylation and reversed at mitotic exit by the timely and ordered dephosphorylation of mitotic substrates. This phosphorylation results from the balance between the activity of kinases and phosphatases. The role of kinases in the control of mitosis has been largely studied, however, the impact of phosphatases has long been underestimated. Recent data have now established that the regulation of phosphatases is crucial to confer timely and ordered cellular events required for cell division. One major phosphatase involved in this process is the phosphatase holoenzyme PP2A-B55. This review will be focused in the latest structural, biochemical and enzymatic insights provided for PP2A-B55 phosphatase as well as its regulators and mechanisms of action.

## Introduction

Protein phosphorylation is a post-translational modification used by the cell to modify protein properties. This modification is essential for the control of cellular processes including mitosis. Entry into mitosis is triggered by the activation of the kinase Cyclin B/Cdk1 that phosphorylates a myriad of proteins and promotes profound physical cellular remodeling. Together with kinases including Plk1, Aurora A, Aurora B, Haspin, Mps1 or Bub1, Cylcin B/Cdk1 specifically and timely phosphorylate different substrates, modify their function/localization and promote essential cellular changes such as nuclear envelope breakdown, chromatin condensation and spindle formation (Nigg, 2001; Lindqvist et al., 2009). These kinases further modulate kinetochore-microtubule attachment and activate a checkpoint mechanism called the Spindle Assembly Checkpoint (SAC) (Lara-Gonzalez et al., 2012; Musacchio, 2015). The SAC prevents chromosome segregation before kinetochores are correctly attached to the microtubules of the spindle and its satisfaction defines a point of no-return from which cells are forced to exit mitosis. This commitment point is marked by the activation of the E3 ubiquitin ligase Anaphase Promoting Complex (APC) that ubiquitinates, among others, the protein Cyclin B and induces its degradation, definitively inactivating Cyclin B/Cdk1 and making mitotic exit irreversible. Cyclin B degradation triggers a specific and progressive program of protein dephosphorylation that will establish the correct temporal pattern of events required for the reformation of a G1 cell (McCloy et al., 2015; Cundell et al., 2016; Swaffer et al., 2018; Touati et al., 2018). Besides Cyclin B proteolysis, the establishment of a fine-tuned program of dephosphorylation requires the activity of several phosphatases that will be sequentially turned on as Cyclin B/Cdk1 becomes inactivated.

Unlike kinases, protein phosphatases have been considered for a long time as housekeeping enzymes with constant activity. However, data from the last years have established the prominent role of the modulation of these enzymes in the control of protein function and cell signaling. Phosphatases such as PP1, PP2A, PP4 or PP6 have been shown to play a primordial role in the control of mitotic progression (Mayer-Jaekel et al., 1994; Chen et al., 2007; Schmitz et al., 2010; Wurzenberger et al., 2012).

In this review, we will focus on one major phosphatase, PP2A. We will first report the major structural features, catalytic mechanisms and substrate recognition of all PP2A holoenzymes. We will next focus on PP2A-B55, the main PP2A holoenzyme responsible for the dephosphorylation of Cyclin B/Cdk1 substrates and key to promote mitotic exit.

### PP2A holoenzymes: Structure and substrate recognition

PP2A is a serine/threonine phosphatase. Serine/Threonine phosphatases are composed of three different families: (1) PhosphoProtein Phosphatases (PPP), (2) Protein Phosphatase Metal-depending (PPM) and (3) FCP/SCP aspartate-dependent phosphatases. The two first families display two metal ions in the catalytic center required for the activation of a water molecule that will mount a nucleophilic attack in the phosphate group and will catalyze dephosphorylation in a single step (Barford et al., 1998). The third family uses an aspartate-based catalysis mechanism and depends on the formation of a phosphoaspartate intermediate (Kamenski et al., 2004).

The PPP are the most abundant phosphatases in the cell. This family includes PP1, PP2A, PP2B (or calcineurin), PP4, PP5, PP6, and PP7 (Shi, 2009). PP2A is the most abundant and represents 1% of the total protein in the cell. This enzyme is composed of three different subunits, a catalytic subunit or PP2A-C, a scaffold subunit or PP2A-A and a regulatory subunit or PP2A-B, the last one conferring substrate specificity. Two different α and β isoforms exist for the PP2A-A and PP2A-C subunits whereas the PP2A-B subunits comprise four groups: (1) B or B55, (2) B′ or B56, (3) B″ or PR48/PR70/PR72/PR130 and (4) B’’’ or Striatins. Each group displays different isoforms and/or members, with isoforms *α, β, γ* and *δ* for the B subunit (Mayer et al., 1991; Strack et al., 1999), *α, β, γ, δ* and *ε* for the B’ (McCright and Virshup, 1995; Csortos et al., 1996), α (PR130/PR72), β (PR48/PR70), and γ (G5PR) for B’’ (Hendrix et al., 1993; Yan et al., 2000; Zwaenepoel et al., 2008) and the Striatin, SG2NA and Zinedin members for the B’’’ some of which also display different isoforms (Castets et al., 1996, 2000).

**FIGURE 1:**
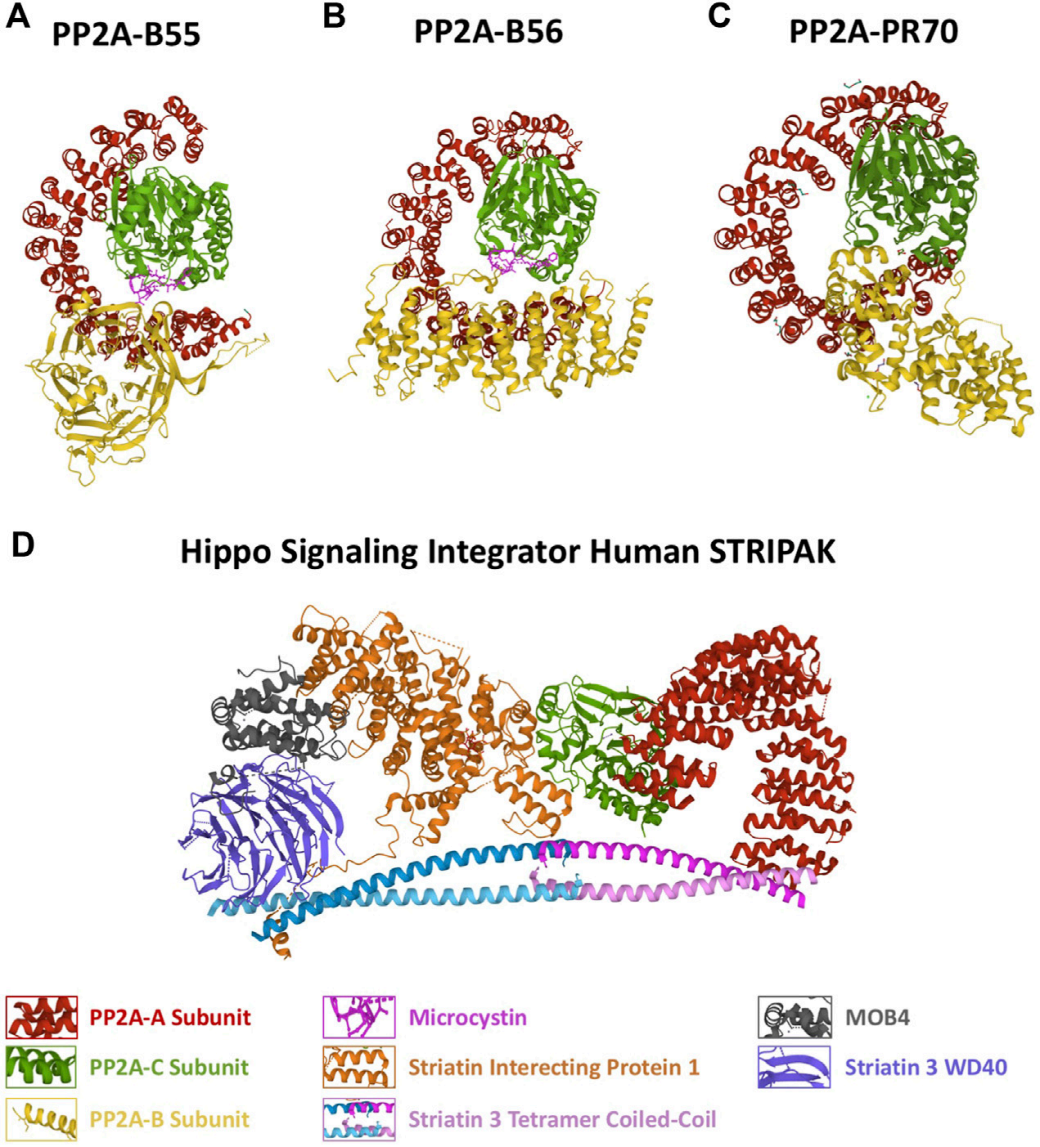
Structure of the different PP2A holoenzymes. Protein structure represented as a cartoon of the **(A)** PP2A-B55, **(B)** PP2A-B56, **(C)** PP2A-PR70 holoenzymes and of the **(D)** Hippo Signaling Integrator Human STRIPAK complex. The different PP2A subunits are represented in different colors as indicated. PDB identifiers: 3DW8/PP2A-B55; 2NYM/PP2A-B56; 4I5L/PP2A-PR70; 7K36/STRIPAK.

The catalytic subunit of PP2A adopts a α/β fold structure typical of the PPP family of phosphatases and contains two metal ions in the catalytic site. The scaffold subunit is formed of 15 HEAT repeat domains, with each HEAT repeat comprising a pair of antiparallel αhelices laterally packed to give rise to a horseshoe shaped structure. The A subunit binds to the catalytic subunit at its C-terminus (11–15 HEAT repeats) (Xing et al., 2006) (Figure 1).

The B (B55) subunit is composed of WD40 repeats forming a seven-bladed β propeller with each blade comprising four antiparallel β strands with an acidic groove in the middle. In the blade 2, β strands extend out of the propeller forming a β hairpin arm that binds to the A scaffold subunit at HEAT repeats 3–7 (Xu et al., 2008). This subunit makes very few interactions with the C catalytic subunit (Figure 1A).

The B’ (B56) does not share structural similarity with B55. This subunit is comprised of eight HEAT-like repeats forming a curved shape resembling PP2A-A with the concave negative charged surface facing the C subunit and the convex hydrophobic surface facing the A scaffolding subunit. Unlike the B55 subunit, B56 display several interactions with the catalytic subunit of the holoenzyme (Xu et al., 2006) (Figure 1B).

The B’’ (PR48/PR70/PR72/PR130) is an elongated α helix protein with two EF hand calcium-binding domains (EF1 D911-D922 and EF2 D985-D996; human B” sequence) and a N-terminal hydrophobic region. The second EF hand calcium-binding motif and the hydrophobic region directly contacts the A subunit whereas one helix at the C-terminus interacts with the C subunit close to the catalytic site (Wlodarchak et al., 2013). In the PP2A-PR70 holoenzyme, the A subunit adopts a compact conformation due to the tripartite binding to PR70 and C resulting in a wider enzyme than PP2A-B55 and PP2A-B56 (Figure 1C).

A main structural difference between this three PP2A holoenzymes lies in the compaction of the scaffold subunit. The PP2A-B55 holoenzyme displays the less compacted A subunit due to a loose binding of PP2A-B to the scaffold subunit and few interactions with PP2A-C. A much more compact conformation of PP2A-A is observed in PP2A-B56, in which besides the interaction of B56 to the scaffold subunit, an additional association of the regulatory subunit to PP2A-C is present. Finally, PP2A-PR70 displays the highest compacted PP2A-A resulting from a double binding with PP2A-A and a single tight interaction with PP2A-C (Wlodarchak et al., 2013) (Figure 1C).

Finally, the last holoenzyme PP2A-B‴ is an atypical PP2A that forms part of a multicomplex named Striatin-Interacting Phosphatase and Kinase (STRIPAK) (Glatter et al., 2009; Ribeiro et al., 2010). B’’’ (Stiatrin) directly associates and put in close proximity the phosphatase PP2A A/C with kinases and other cell signaling proteins promoting its dephosphorylation. As for the other PP2A complexes, B’’’ (Striatin) directly interacts with the A scaffold subunit. These B regulatory proteins display four protein-interaction domains including a calmodulin-binding domain, a caveolin-binding domain, a coiled-coil domain and a WD-repeat domain (Castets et al., 1996; Gaillard et al., 2001). The coiled-coil domain of Striatin forms an asymmetric homodimer. Two striatin dimers interact head-to-head through their N-terminal end to form an anti-parallel tetramer region that directly binds to the PP2A-A dimer by its N-terminus whereas not contact exits with the PP2A-C subunit (Figure 1D) (Chen et al., 2014; Jeong et al., 2021).

The B regulatory subunits are responsible of substrate recognition by PP2A holoenzymes. This recognition is provided by the specific structural properties of each B subunit and its interaction with PP2A-A/C. Recent data identified the mechanisms involved in substrate recognition for the PP2A-B55 and PP2A-B56 holoenzymes. These data support the identification by the B regulatory subunit of specific Short Linear interaction Motifs or SLiMs in the substrate sequence, a mechanism already shown for other phosphatases such as PP1 (Roy and Cyert, 2009; Bollen et al., 2010). The presence of SLiMs for PP2A-B56 was first identified in BubR1 and RepoMan proteins (Qian et al., 2013). A subsequent study permitted to attribute the sequence “LxxIxE” as a consensus motif (Hertz et al., 2016; Wang et al., 2016). The LxxIxE SLiM is usually present within disordered regions and associates to a hydrophobic pocket on the B56 subunit between HEAT repeats 3 and 5. This binding is enhanced by the presence of a phosphorylated site inside or immediately C-terminally to the motif (Hertz et al., 2016).

First data on PP2A-B55 substrate recognition revealed the preference of the phosphatase towards phospho-Thr versus phospho-Ser (McCloy et al., 2015) and a prominent role of basic amino acids (preferentially Lysin and Arginine amino acids) upstream and downstream the phosphorylated site (Cundell et al., 2016). As for PP2A-B56, a recent study has attributed a SLiM consensus sequence “pSPxxHxRVxxV” for PP2A-B55. In this motif B55-substrate association would be mediated by the interaction of the H and R amino acids of the SLiM with D197 of B55α (Fowle et al., 2021).

The discovery of SLiMs for PP2A-B55 and PP2A-B56 prompted the interrogation of human full proteome for the identification of proteins containing these SLiM consensus motifs that could eventually be dephosphorylated by these two enzymes (Hertz et al., 2016; Wu et al., 2017; Fowle et al., 2021). This approach permitted the identification of SLiMs in already known substrates for these phosphatases and supply a large list of other putative substrates that will undoubtably provide new interesting insides in the role of these enzymes in cell signaling.

### PP2A-B55 in the control of mitosis: The Greatwall/Arpp19-ENSA/PP2A-B55 axis

Data demonstrating the essential role of the control of phosphatases in mitotic progression was first obtained in the *Xenopus* egg extract model. These data unambiguously identified PP2A-B55 as the phosphatase counterbalancing Cyclin B/Cdk1 during interphase and demonstrated that its inhibition is essential for the correct timing of protein phosphorylation during mitosis (Mochida et al., 2009). Simultaneously, another study demonstrated a role of the kinase Greatwall (Gwl) in the control of mitotic progression *via* the negative modulation of PP2A-B55 (Vigneron et al., 2009). These data have been supported by subsequent reports showing that mitosis is profoundly impacted by the missregulation of Gwl or PP2A-B55 (Castilho et al., 2009; Burgess et al., 2010; Voets and Wolthuis, 2010; Kim et al., 2012; Alvarez-Fernandez et al., 2013; Cundell et al., 2013).

**FIGURE 2:**
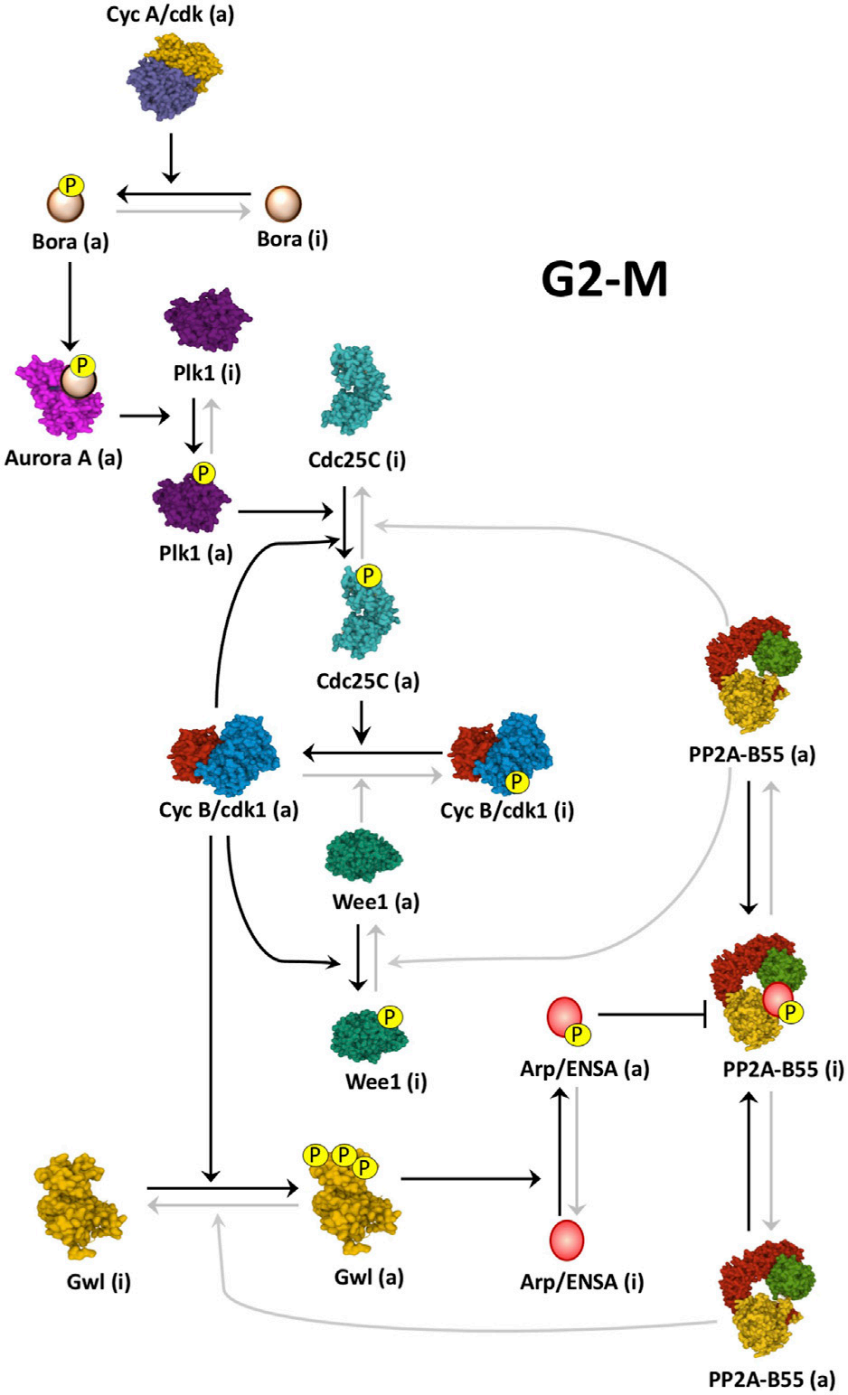
Cyclin B/Cdk1 activation loop at mitotic entry. Schematic of how Cyclin B/Cdk1 and the Gwl/Arpp19-ENSA/PP2A-B55 cascades are activated at mitotic entry. At G2-M, Cyclin A/Cdk activity triggers the Cyclin B/Cdk1 amplification loop and the Gwl/Arpp19-ENSA/PP2A-B55 cascade by promoting the phosphorylation of Bora and the subsequent activation of Plk1 by Aurora A. Black arrows represent active pathways inducing mitotic entry. Linking line finishing with arrowheads: activation. Linking line finishing with perpendicular bar: inhibition. Black lines: major active pathways at G2-M. Gray lines: inactive pathways at G2-M. (a): active; (i): inactive. PDB identifiers: 3OP3/Cdc25; 4BYJ/Aurora A; 6GUF/CyclinA-Cdk2; 3D5U/Plk1; 5LOH/Gwl; 6GU4/CyclinB-Cdk1; 5VD2/Wee1; 3DW8/PP2A-B55.

Gwl activation at mitotic entry is essential for correct cell division. This kinase is activated by the phosphorylation on two residues, Thr194 and Thr207 (human Gwl sequence) on its catalytic site by Cyclin B/Cdk1, and its subsequent autophosphorylation at its C-terminal tail on Ser875 (Vigneron et al., 2011; Blake-Hodek et al., 2012). Once active, Gwl phosphorylates its substrates Arpp19 and ENSA, two intrinsically disordered proteins that when phosphorylated, potently bind and inhibit PP2A-B55 (Gharbi-Ayachi et al., 2010; Mochida et al., 2010) (Figure 2). Inhibition of PP2A-B55 permits the stable phosphorylation of mitotic substrates and mitotic progression (Gharbi-Ayachi et al., 2010; Mochida et al., 2010; Rangone et al., 2011; Larouche et al., 2021). Intriguingly, Gwl/Arpp19-ENSA/PP2A-B55 axis is essential for mitotic entry and maintenance in *Xenopus* oocytes (Vigneron et al., 2009; Gharbi-Ayachi et al., 2010; Mochida et al., 2010), however, although mitotic progression is highly impacted, the knockout of the mouse Greatwall gene *Mastl* or of the *Arpp19* gene do not prevent Mouse Embryonic Fibroblast (MEFs) from entering mitosis (Alvarez-Fernandez et al., 2013; Hached et al., 2019). Although apparently contradictory, these data could be explained by either a different balance between Cyclin B/Cdk1/PP2A-B55 in these two models or by the presence of an additional negative modulator of this phosphatase that could participate to its inhibition at G2/M transition.

Once in anaphase, the gradual inactivation of the Gwl/Arpp19-ENSA/PP2A-B55 axis is essential for the cell to exit mitosis. The progressive reactivation of PP2A-B55 organizes the temporal pattern of protein dephosphorylation and the programmed cellular events required for cell division (Burgess et al., 2010; Voets and Wolthuis, 2010; Alvarez-Fernandez et al., 2013; Cundell et al., 2013; McCloy et al., 2015; Hached et al., 2019). This reactivation is triggered at anaphase by the APC-dependent ubiquitination and degradation of Cyclin B that results in a gradual drop of Cyclin B/Cdk1 activity, in the dephosphorylation of Gwl and Arpp19/ENSA and in the progressive reactivation of PP2A-B55.

### Spatiotemporal regulation of the Greatwall/Arpp19-ENSA/PP2A-B55 axis during mitosis

The Gwl/Arpp19-ENSA/PP2A-B55 axis is switched on at G2-M. This transition is triggered by the mutual control of Cyclin B/Cdk1 and Gwl kinases. At mitotic entry, Cyclin B/Cdk1 phosphorylates and positively modulates Gwl (Vigneron et al., 2011; Blake-Hodek et al., 2012), whereas Gwl, *via* phospho-Arpp19-ENSA and PP2A-B55 inhibition, maintains the phosphorylation of Wee1/Myt1 and Cdc25, three PP2A-B55 substrates and key enzymes modulating Cyclin B/Cdk1 activity (Zhao et al., 2008; Vigneron et al., 2009; Lorca et al., 2010) (Figure 2).

During G2, Cyclin B/Cdk1 is maintained repressed by the phosphorylation of its Cdk1 subunit on residues Thr14 and Tyr15 (Draetta and Beach, 1988; Gould and Hunter, 1988; Krek and Nigg, 1991). These phosphorylations are induced by the Wee1/Myt1 kinases (Parker and Piwnica-Worms, 1992; Mueller et al., 1995) and removed by the Cdc25 phosphatase (Gautier et al., 1991; Kumagai and Dunphy, 1991; Strausfeld et al., 1991). At G2-M transition, a partial activation of Cdc25 promotes the dephosphorylation and activation of a pool of Cyclin B/Cdk1. This pool will phosphorylate Wee1/Myt1 and Cdc25 promoting the negative modulation of the inhibitory kinases and the positive modulation of the activatory phosphatase and inducing full Cyclin B/Cdk1 activation and mitotic entry. This mechanism has been called the Cyclin B/Cdk1 amplification loop (Izumi et al., 1992; Hoffmann et al., 1993). Although reported a long time ago, the key question of how Cdc25 is partially activated to trigger this feedback loop was not known. This issue has only been recently resolved by two groups that showed that Cyclin A/Cdk1/2 is the missing piece of the puzzle. These groups demonstrated that the main kinase triggering mitotic entry is Cyclin A/Cdk1/2 (Vigneron et al., 2018; Hégarat et al., 2020). At late G2, the activity of this kinase rises and phosphorylates the protein Bora that promotes Aurora A-dependent activation of Plk1. Once active, Plk1 partially phosphorylates Cdc25 resulting in the activation of a pool of Cyclin B/Cdk1 (Vigneron et al., 2018). This active pool rapidly triggers the amplification loop and phosphorylates and activates Gwl. Phospho-Arpp19/ENSA then inhibit PP2A-B55, preventing Wee1/Myt1/Cdc25 dephosphorylation and ensuring full Cyclin B/Cdk1 activation (Figure 2). These interconnected loops create bistability and confer robustness, irreversibility and a switch-like behavior to mitotic entry, three properties that are essential to prevent intermediate interphase-mitotic states that could be very detrimental to the cell (Hegarat et al., 2014; Mochida et al., 2016; Hutter et al., 2017; Rata et al., 2018).

**FIGURE 3:**
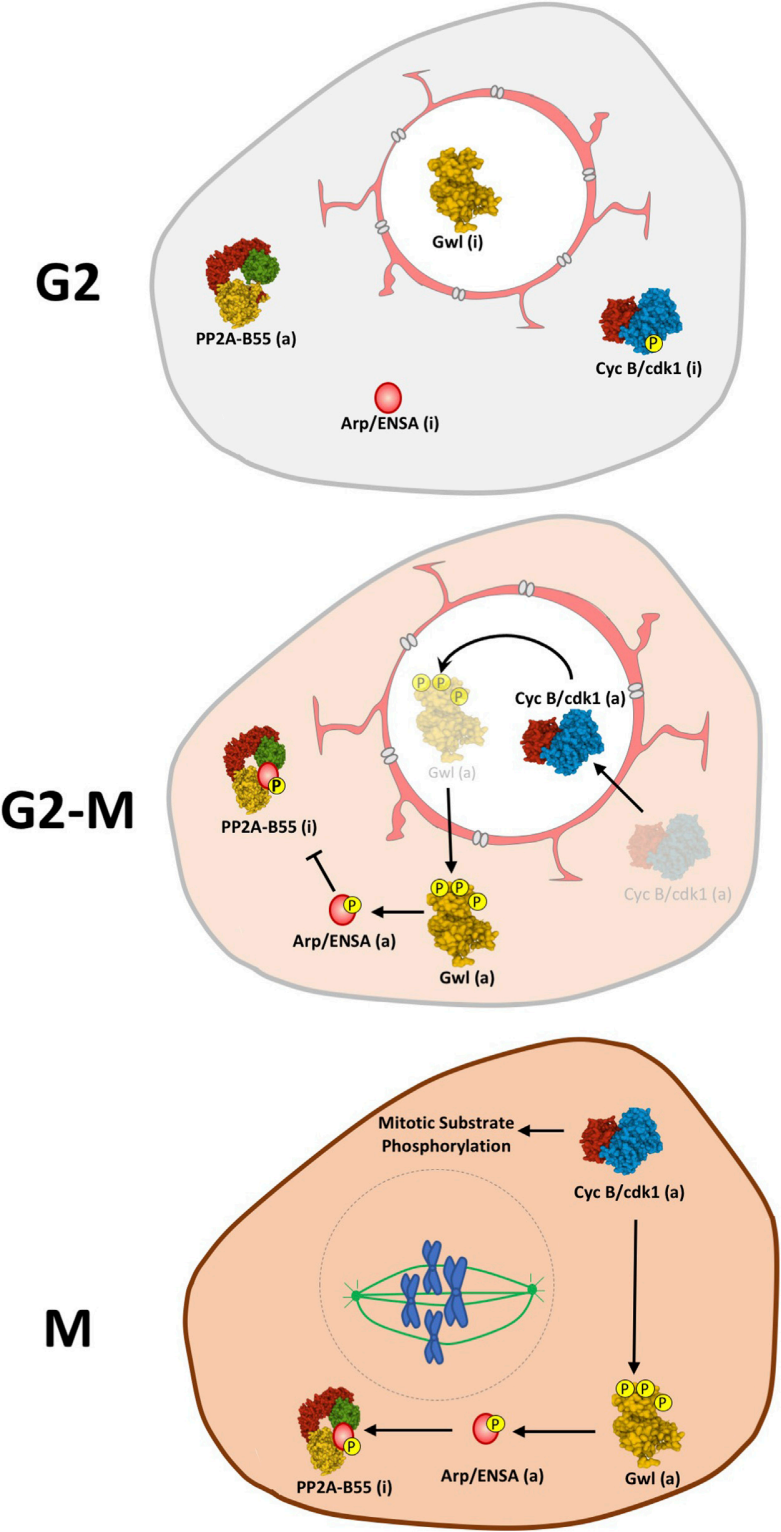
Model for the spatiotemporal regulation of the Gwl/Arpp19-ENSA/PP2A-B55 axis. During G2 Gwl kinase is maintained inactive in the nucleus while Cyclin B/Cdk, ENSA and PP2A-B55 are present in the cytoplasm. At G2-M, CyclinB-Cdk1 is activated and relocalized in the nucleus where it will phosphorylate and activate Gwl. Once activated, Gwl will translocate to the cytoplasm where it will subsequently phosphorylate Arpp19/ENSA triggering its binding to PP2A-B55 and the inhibition of this phosphatase. The drop of PP2A-B55 phosphatase activity will now allow the stable phosphorylation of mitotic substrates and maintain mitosis. Linking line finishing with arrowheads: activation. Linking line finishing with perpendicular bar: inhibition. (a): active; (i): inactive.

Beside its molecular regulation, the activation of the Gwl/Arpp19-ENSA/PP2A-B55 axis is also tightly controlled by subcellular localization. To ensure the activation of these two interlinked loops, both Cyclin B/Cdk1 amplification loop and Gwl/Arpp19-ENSA/PP2A-B55 axis have to co-localize at G2-M entry (Figure 3). Cyclin B is imported into the nucleus at G2-M transition where it activates the amplification loop (Lindqvist et al., 2009; Gavet and Pines, 2010; Santos et al., 2012). Gwl is a nuclear protein that becomes cytoplasmic in prophase (Alvarez-Fernandez et al., 2013; Wang et al., 2013). The nuclear localization of this kinase is mediated by the presence of two NLS sequences and its cytoplasmic exclusion appears to be mediated by the phosphorylation of these motifs. So far, two different kinases, Cyclin B/Cdk1 in MEFs (Alvarez-Fernandez et al., 2013) and Plk1 in the *Drosophila* model (Wang et al., 2013) have been proposed as responsible of this phosphorylation. Unlike Gwl, B55 is localized in the cytoplasm throughout the cell cycle (Mayer-Jaekel et al., 1994; Santos et al., 2012; Larouche et al., 2021). Finally, different localizations have been reported for ENSA (Endos in *Drosophila*), being cytoplasmic for the *Drosophila* Endos (Larouche et al., 2021) and nuclear/cytoplasmic in human cells (Charrasse et al., 2017). The current model proposes that, at the G2/M transition, the colocalization of Gwl with Cyclin B/Cdk1 in the nucleus, away from PP2A-B55, will permit their activation. At prophase, before nuclear envelope breakdown, activated Gwl will move to the cytoplasm where it will phosphorylate ENSA and inhibit PP2A-B55 (Alvarez-Fernandez et al., 2013; Wang et al., 2013; Larouche et al., 2021). However, since as reported below, Gwl is a PP2A-B55 substrate (Heim et al., 2015; Ma et al., 2016; Rogers et al., 2016; Ren et al., 2017), this model does not explain how Gwl phosphorylation and activity is maintained in the cytoplasm to induce ENSA phosphorylation in the presence of a fully active phosphatase. Alternatively, ENSA can be partially localized in the nucleus, as previously described for human ENSA (Charrasse et al., 2017), where a pool of this protein would be phosphorylated by Cyclin B/Cdk1-activated Gwl. Once phosphorylated, ENSA would be transported into the cytoplasm where it would inhibit PP2A-B55. Partially inhibited PP2A-B55 holds cytoplasmic Gwl activity and maintains ENSA phosphorylation and phosphatase inhibition.

**FIGURE 4:**
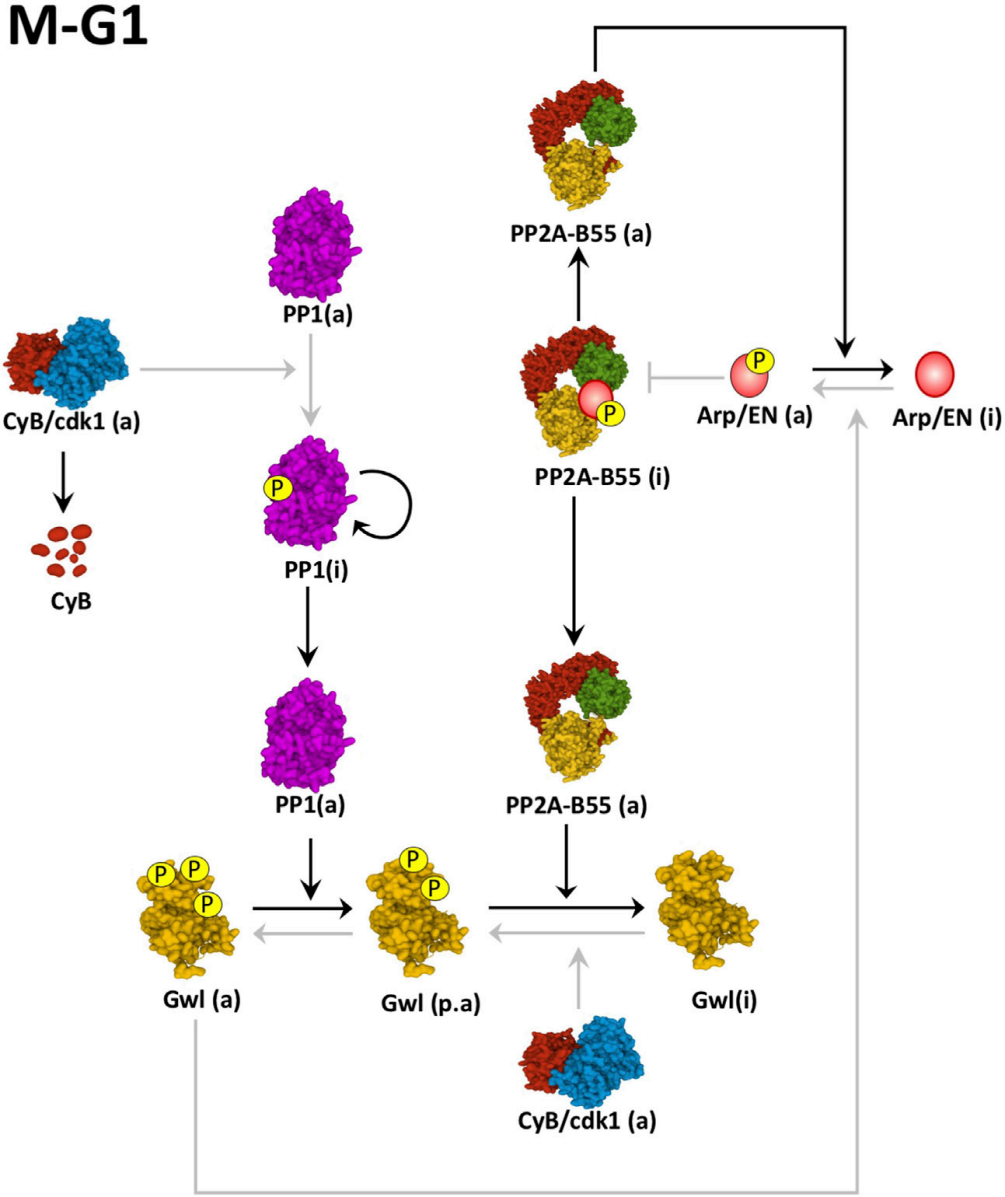
Mechanisms of Gwl inactivation at mitotic exit. Cyclin B degradation results in auto-dephosphorylation of PP1. Active PP1 promotes the dephosphorylation of Gwl on its Ser875 providing to this Gwl a partial activity (p.a). The partial inactivation of this kinase results in the intermediate reactivation of PP2A-B55, that promotes the full dephosphorylation of Gwl and Arpp19-ENSA (Arp/EN) and mitotic exit. Black arrows represent active pathways inducing mitotic exit. Grey arrows represent inactive pathways. (a): active; (i): inactive. Linking line finishing with arrowheads: activation. Linking line finishing with perpendicular bar: inhibition. Black lines: major active pathways at M-G1. Gray lines: inactive pathways at M-G1. PDB identifiers: 6GU4/CyclinB-Cdk1; 4MOV/PP1; 5LOH/Gwl; 3DW8/PP2A-B55.

Cyclin B/Cdk1 and Gwl activity will be maintained high until anaphase when the activation of the APC will induce the progressive ubiquitination and degradation of Cyclin B. Cyclin B/Cdk1 complex does not only directly phosphorylate Gwl to maintain its activity but also phosphorylates and inhibits PP1, the phosphatase responsible of the dephosphorylation of Gwl on its Ser875 C-tail residue (Figure 4). At anaphase, the progressive decrease of Cyclin B/Cdk1 activity ensues with a gradual autodephosphorylation and reactivation of PP1 (Wu et al., 2009). Active PP1 induces dephosphorylation of Gwl on Ser875 and decreases phospho-Arpp19-ENSA enabling the activation of a threshold level of PP2A-B55 that will subsequently dephosphorylate Gwl on its Thr194-Thr207 T-loop activatory sites. Gwl will be now completely inactivated and will trigger a negative feedback loop resulting in the overall reactivation of PP2A-B55 and mitotic exit (Heim et al., 2015; Ma et al., 2016; Rogers et al., 2016; Ren et al., 2017). Besides PP1 and PP2A-B55, FCP1 also dephosphorylates Gwl on other Cyclin B/Cdk1 phosphosites including Ser90 and S453, however, the exact role of these sites in the control of kinase activity has not been reported (Della Monica et al., 2015).

### PP2A-B55 inhibition by Arpp19/ENSA

Arpp19 and ENSA are two intrinsically unstructured proteins from the endosulfine family reported so far as the two unique substrates of the Gwl kinase (Gharbi-Ayachi et al., 2010; Mochida et al., 2016). Besides Gwl, these two proteins can also be phosphorylated and modulated by two other kinases: Cyclin B/Cdk1 and PKA. Cyclin B/Cdk1 phosphorylates Arpp19/ENSA at its N-terminus on residues Ser23/Ser21 (human Arpp19/ENSA sequence respectively). *In vitro* assays demonstrate that phospho-Ser21-ENSA displays PP2A-B55 inhibitory activity although it only represents 14-fold inhibitory potential compared to 3800-fold inhibitory potential for the ENSA protein phosphorylated by Gwl (Mochida, 2014). However, despite this *in vitro* inhibitory activity, whether the phosphorylation of this site plays a physiological role is completely unknown so far.

Arpp19/ENSA main phosphorylation site is Ser62/Ser67 (human Arpp19/ENSA sequence respectively). This residue is phosphorylated by the Gwl kinase and is the essential site controlling Arpp19/ENSA association to PP2A-B55 (Gharbi-Ayachi et al., 2010; Mochida et al., 2010). Recent studies addressed the mechanisms governing Arpp19/ENSA-PP2A-B55 binding and inhibition. These studies demonstrate that Arpp19/ENSA are indeed substrates of PP2A-B55 and that their phosphorylation by Gwl increases their affinity towards the phosphatase around 4000-fold. However, their dephosphorylation is very slow with a K_Cat_ around 0.030 s^−1^ compared to a K_Cat_ of 25 s^−1^ for the other regular substrates of the phosphatase. Thus, phospho-Arpp19/ENSA inhibit PP2A-B55 by competing with their substrates (Williams et al., 2014). Data also demonstrate that the inhibition of PP2A-B55 by Arpp19/ENSA requires Ser62/Ser67 adjacent residues included in the sequence ^59^YFDSGD^64^ (of human Arpp19 sequence). The mutation into alanine of any of these residues in a phospho-Ser62 Arpp19 form induces a rapid dephosphorylation of its Gwl site with close kinetics to those observed for regular PP2A-B55 substrates indicating that they are not competing anymore and consequently, that they lost their inhibitory capacity (Labbé et al., 2021). However, although these residues are essential for PP2A-B55 inhibition, whether they participate to the interaction with the PP2A-B55 heterocomplex is completely unknown so far. In this regard, a role in the interaction with acidic residues on the B55 subunit has been proposed for basic Lys/Arg residues flanking the sequence containing the Gwl phosphorylation site (DSG motif) (Cundell et al., 2016), however, mutation of these residues appears not to impact their dephosphorylation kinetics and their PP2A-B55 inhibitory activity (Labbé et al., 2021).

Arpp19/ENSA can be also phosphorylated on Ser109/Ser112 (human Arpp19/ENSA sequence respectively) by PKA. This phosphorylation is essential to maintain prophase arrest in oocytes and to disable the formation of a critical threshold of Cyclin B/Cdk1 activity required for meiotic resumption (Dupre et al., 2013; Dupré et al., 2014; Lemonnier et al., 2021). Phospho-Ser109/Ser112 delays the stably phosphorylation of the Gwl site by increasing its dephosphorylation and confers, in this way, the correct temporal pattern of Arpp19/ENSA inhibitory activity (Labbé et al., 2021).

Both, Arpp19 and ENSA inhibit PP2A-B55 with similar kinetics. The question that emerges is then, why the cell requires two different inhibitors of this phosphatase? The answer was recently provided by comparing the phenotypes of Arpp19 and ENSA knockout (KO) mice. Arpp19 knockout revealed to be lethal. Upon *Arpp19* KO, embryonic development arrested at gastrulation with cells displaying a high mitotic index. Further analysis in Arpp19 KO MEFs demonstrated that the ablation of this gene promotes dramatic mitotic defects that result in increased cell death and aneuploidy (Hached et al., 2019). Conversely, the KO of the *ENSA* gene does not perturb early embryonic development suggesting that this protein is differentially controlling cell proliferation. Indeed, accordingly, the knockdown of this protein in human cells induces DNA replication defects by decreasing the number of active replication forks (Charrasse et al., 2017). Thus, the presence of two PP2A-B55 inhibitors confers to the cell a means to fine-tune this phosphatase under different cellular contexts.

### PP2A-B55-dependent dephosphorylation and mitotic exit

Once chromosomes are correctly attached to the fibers of the spindle, the cell exits mitosis. This is preceded by the silencing of the SAC and the subsequent activation of the APC that will in turn, induce the sequential degradation of Cyclin B, the dephosphorylation of Gwl and of Arpp19/ENSA, and the reactivation of PP2A-B55. Active PP2A-B55 will then promote a sequential dephosphorylation of mitotic substrates that will ensure the ordered cellular events conducting cell division. Chromosome segregation, cytokinesis furrow formation, chromatin decondensation or nuclear envelop reformation are promoted by the precise temporal pattern of PP2A-B55-dependent substrate dephosphorylation. Modifying this pattern for example, by knocking down/out Gwl, Arpp19 or B55 profoundly impacts cell division by perturbing the order of the different cellular events (Burgess et al., 2010; Voets and Wolthuis, 2010; Alvarez-Fernandez et al., 2013; Cundell et al., 2013; Hached et al., 2019). Accordingly, in Gwl knockdown and Arpp19 knockout cells, DNA decondensation is observed in some of these cells before chromosome alignment, cytokinesis furrow formation often precedes sister chromatid segregation and nuclear envelope can be reformed before the end of anaphase (Burgess et al., 2010; Hached et al., 2019).

Recent data obtained by global phosphoproteomic studies have identified new PP2A-B55 substrates and provided new insights in the role of PP2A-B55-dependent dephosphorylation in mitotic exit (McCloy et al., 2015; Cundell et al., 2016; Godfrey et al., 2017; Touati et al., 2018; Holder et al., 2020).

One of these substrates is the SAC protein Mps1 (Diril et al., 2016). At prometaphase, SAC activation will be mediated by the localization of Mps1 at unattached kinetochores where it will become activated and will phosphorylate the kinetochore protein KNL1 and the checkpoint proteins Bub1 and Mad1 (Ciliberto and Hauf, 2017). Clustering of Mps1 molecules in unattached kinetochores induces T-loop autophosphorylation and turns on this kinase (Kang et al., 2007) whereas its additional phosphorylation by Cyclin B/Cdk1 is required to keep its activity (Morin et al., 2012). At anaphase onset, once microtubules of the spindle are correctly attached to kinetochores, Mps1 is evicted from these chromosomal structures (Hiruma et al., 2015; Ji et al., 2015) and submitted to T-loop dephosphorylation by PP2A-B56 (Hayward et al., 2019). Interestingly, recent data indicate that, besides the T-loop, the correct temporal pattern of dephosphorylation of the Cyclin B-Cdk1-dependent sites of Mps1 by PP2A-B55 is also essential for normal mitotic exit. Accordingly, the inappropriate reactivation of this phosphatase by Gwl KO in MEFs results in a premature dephosphorylation of Mps1 and SAC silencing resulting in perturbed mitotic exit, a phenotype that can be rescued by the inhibition of PP2A by okadaic acid (Diril et al., 2016).

Some constituents of the APC have also been identified as substrates of PP2A-B55. At early mitosis, Cyclin B/Cdk1, phosphorylates the structural constituent Apc1 on Ser355 and the regulatory subunit Cdc20 on Thr70. Phosphorylation of Apc1 is essential to permit the binding of Cdc20 to the APC complex that will trigger its activation (Zhang et al., 2016). Conversely, for Cdc20, the association to the APC requires its dephosphorylation (Labit et al., 2012; Diril et al., 2016; Lee et al., 2017). Thus, for the APC to be activated, Cdc20 dephosphorylation must precede the one of Apc1 (Hein et al., 2017). PP2A-B55 has an inherent preference for phospho-Thr versus phospho-Ser and this could explain the ordered dephosphorylation of these two APC components. Indeed, compelling data point to a role of this phosphatase in Cdc20 and Apc1 dephosphorylation. The knockdown of B55 in Hela cells as well as the Thr-to-Ser mutation of Cyclin B/Cdk1 phosphorylation site of Cdc20 perturbs the pattern of dephosphorylation of this protein and mitotic exit (Hein et al., 2017). Moreover, the KO of Arpp19 in MEFs results in a too rapid inactivation of the APC and the incomplete degradation of Cyclin B (Hached et al., 2019). However, although it can participate to establish the gradual dephosphorylation of Apc1 and Cdc20, PP2A-B55 cannot be the phosphatase triggering APC activation since its reactivation requires Cyclin B degradation and Gwl/Arpp19-ENSA dephosphorylation. Instead, this role could be played by either PP2A-B56 (Lee et al., 2017) or PP1 (Kim et al., 2017), two phosphatases already active in the kinetochore from early mitosis.

Once activated, the APC triggers anaphase and chromosome segregation. Subsequently, a structure referred as the central spindle is formed on antiparallel microtubules. This structure, key to define the position of the cytokinesis furrow, is induced by the recruitment at the center of the spindle of the bundling protein PRC1. PRC1 is phosphorylated by Cyclin B/Cdk1 on Thr 470 and 481 at early mitosis and this phosphorylation prevents its binding to the microtubules (Jiang et al., 1998; Mishima et al., 2004; Zhu et al., 2006). Upon Cyclin B degradation and Gwl/Arpp19-ENSA dephosphorylation, PP2A-B55 is reactivated inducing the dephosphorylation of PRC1 (Cundell et al., 2013; Hached et al., 2019). Dephosphorylated PRC1 then binds antiparallel microtubules and promotes anaphase spindle elongation and central spindle formation (Zhu et al., 2006; Walczak and Shaw, 2010).

Finally, to return to interphase cells have to reform the nuclear envelope and the nuclear pore complexes. The nuclear envelope is composed of an outer and an inner nuclear membrane. The inner membrane contains transmembrane proteins of the LEM domain family that contact with lamins, a filamentous meshwork of intermediate filaments underlying the nuclear envelope (Burke and Ellenberg, 2002) and with BAF, a chromatin-binding protein that links nuclear envelope with chromatin (Wagner and Krohne, 2007). At early mitosis, nuclear pore proteins, lamins and BAF are phosphorylated, by Cyclin B/Cdk1 for the first two proteins (Peter et al., 1990, 1990; Glavy et al., 2007; Laurell et al., 2011) and by the VRK kinase for the third one (Nichols et al., 2006) and this phosphorylation causes nuclear envelope disassembly. At the end of mitosis, these proteins have to be dephosphorylated to reform the nuclear envelope. This dephosphorylation is dependent on PP2A-B55. Accordingly, the premature activation of PP2A-B55 by either Gwl knockdown in human cells or Arpp19 KO in MEFs promotes the precocious dephosphorylation of these nuclear components and advances nuclear envelope reformation (Cundell et al., 2013; Hached et al., 2019). On the contrary, the partial loss of Tweens (B55 subunit in *Drosophila*) in early embryos of *Drosophila* delays lamin and BAF recruitment to nascent nuclei (Mehsen et al., 2018). Interestingly, major phosphosites in these late dephosphorylated nuclear proteins correspond to Ser, confirming that the preference of this phosphatase for phospho-Thr over phospho-Ser participates to the establishment of the proper order of protein dephosphorylation and confers the correct timing of the mitotic events.

## Concluding remarks

The discovery of the accurate regulation of phosphatases has allowed a great step forward in the understanding of the mechanisms controlling mitosis. New provided structural data, the identification of SliMs controlling phosphatase-substrate interaction and the discovery of new phosphatase inhibitors have significantly increased our knowledge of how the temporal pattern of protein dephosphorylation is established during mitotic exit. However, capital questions remain still unanswered. Notably, although PP2A-B55 could contribute to program dephosphorylations by preferentially selecting phospho-Thr versus phospho-Ser sites, how it temporally orders dephosphorylations of Cyclin B/Cdk1 substrates containing a similar phospho-site is completely unknown. In this line, the nature of the associated SLiM could participate to this order, however, this issue has not been studied yet. Additionally, whether a differential subcellular localization of PP2A-B55 isoforms could participate to the establishment of a temporal pattern of protein dephosphorylation is not known. Other major challenges for the future include the identification of new specific substrates or inhibitors for this phosphatase and the mechanisms by which they are recognized and interact with this enzyme. Finally, the identification of molecules targeting SLiM-phosphatase interaction and substrate dephosphorylation would not only represent a major advance for the study of the function of this enzyme but could additionally provide new interesting therapeutic tools.
